# Population-Genomic Analysis Identifies a Low Rate of Global Adaptive Fixation in the Proteins of the Cyclical Parthenogen *Daphnia magna*

**DOI:** 10.1093/molbev/msac048

**Published:** 2022-03-04

**Authors:** Peter D Fields, Seanna McTaggart, Céline M O Reisser, Christoph Haag, William H Palmer, Tom J Little, Dieter Ebert, Darren J Obbard

**Affiliations:** 1 Department of Environmental Sciences, Zoology, University of Basel, Basel, Switzerland; 2 Institute of Evolutionary Biology; School of Biological Sciences, University of Edinburgh, Edinburgh, United Kingdom; 3 CEFE, Univ Montpellier, CNRS, EPHE, IRD, Montpellier, France; 4 MARBEC, Univ Montpellier, CNRS, IFREMER, IRD, Montpellier, France

**Keywords:** *Daphnia magna*, adaptive evolution, arms race, distribution of fitness effects, McDonald–Kreitman, immune genes, RNA interference

## Abstract

*Daphnia* are well-established ecological and evolutionary models, and the interaction between *D. magna* and its microparasites is widely considered a paragon of the host-parasite coevolutionary process. Like other well-studied arthropods such as *Drosophila melanogaster* and *Anopheles gambiae*, *D. magna* is a small, widespread, and abundant species that is therefore expected to display a large long-term population size and high rates of adaptive protein evolution. However, unlike these other species, *D. magna* is cyclically asexual and lives in a highly structured environment (ponds and lakes) with moderate levels of dispersal, both of which are predicted to impact upon long-term effective population size and adaptive protein evolution. To investigate patterns of adaptive protein fixation, we produced the complete coding genomes of 36 *D. magna* clones sampled from across the European range (Western Palaearctic), along with draft sequences for the close relatives *D. similis* and *D. lumholtzi*, used as outgroups. We analyzed genome-wide patterns of adaptive fixation, with a particular focus on genes that have an *a priori* expectation of high rates, such as those likely to mediate immune responses, RNA interference against viruses and transposable elements, and those with a strongly male-biased expression pattern. We find that, as expected, *D. magna* displays high levels of diversity and that this is highly structured among populations. However, compared with *Drosophila*, we find that *D. magna* proteins appear to have a high proportion of weakly deleterious variants and do not show evidence of pervasive adaptive fixation across its entire range. This is true of the genome as a whole, and also of putative ‘arms race’ genes that often show elevated levels of adaptive substitution in other species. In addition to the likely impact of extensive, and previously documented, local adaptation, we speculate that these findings may reflect reduced efficacy of selection associated with cyclical asexual reproduction.

## Introduction

Estimates of the rate of adaptive protein evolution vary enormously among species (Wright and Andolfatto 2008; Gossmann et al. 2012; Galtier 2016). But, despite substantial theory and empirical data, the primary causes of this variation remain uncertain (Galtier 2016; Rousselle et al. 2020). To a first approximation, the rate of adaptive fixation is expected to correlate with population size (reviewed in Lanfear et al. [2014]). This is because the supply of mutations is greater in larger populations and the impact of genetic drift is reduced (i.e., effective population size, *N*_e_, is larger, though see Rousselle et al. [2020]). With a larger effective population size, even mutations with a very small beneficial effect can spread in response to natural selection. However, an increasing body of empirical data finds no clear relationship between effective population size and the rate of adaptive fixation across species (e.g., Galtier 2016; Kern and Hahn 2018). The absence of this relationship between *N*_e_ and adaptive fixation is in contrast with the increased efficacy of purifying selection in large populations. A reduction in the number of nonadaptive amino-acid substitutions and a relative reduction in nonsynonymous pairwise diversity (*π*_A_) has been shown to be significantly correlated with *N*_e_ (estimated from synonymous diversity, *π*_S_) (Galtier 2016; Chen et al. 2017).

Two explanations for the lack of a relationship between *N*_e_ and the rate of adaptive evolution have been proposed. First, more complex population genetic models suggest the impact of population size is not clear-cut when the relationship between selective interference and the distribution of fitness effects (DFE) is considered (reviewed in Lanfear et al. [2014]), or when the ability of populations to track phenotypic optima is considered (Lourenço et al. 2013; Huber et al. 2017). Second, and inherent to many of the analytical frameworks for quantifying rates of adaptive evolution, is that the approaches used may be inadequate or biased. Most estimates derive from extensions to the McDonald–Kreitman (MK) test for detecting selection (McDonald and Kreitman 1991). These approaches contrast the ratio of nonsynonymous to synonymous fixed differences between species (*D*_N_/*D*_S_) with the same ratio for polymorphisms within species (*P*_N_/*P*_S_) (Smith and Eyre-Walker 2002). In its original formulation this framework can be extended to estimate the number of adaptive amino acid substitutions between species as *a* = *D*_N_ − *D*_S_(*P*_N_/*P*_S_), that is, the observed number of amino acid substitutions (*D*_N_) minus the expected number of nonadaptive substitutions (*D*_S_(*P*_N_/*P*_S_)). This is valid under the assumption that synonymous variants are unconstrained and that segregating alternative nonsynonymous alleles are neutral with respect to each other (Eyre-Walker 2006). A problem with the MK approach is that nonsynonymous alleles that are neither quickly removed by purifying selection nor fixed by positive selection increase *P*_N_ but not *D*_N_, and so give spuriously low or even negative estimates of α (the fraction of nonsynonymous differences driven to fixation by positive selection). This includes alleles under balancing selection and those mediating local adaptation, which tend to remain at intermediate frequencies. It also includes weakly deleterious alleles, which can segregate for an extended period before they are lost. Mitigating the impact of weakly deleterious alleles, and accounting for the presence of segregating beneficial alleles, has driven much of the recent development of the field (Keightley and Eyre-Walker 2007; Charlesworth and Eyre-Walker 2008; Eyre-Walker and Keightley 2009; Schneider et al. 2011; Messer and Petrov 2013; Tataru et al. 2017). Nevertheless, it should be noted that MK-based approaches such as these are not intended to detect local adaptation, adaptive fixation at noncoding sites (though see Keightley et al. 2005; and Williamson et al. 2014 where MK-like tests are applied to noncoding genomic regions), or other forms of adaptive evolution—such as that mediated by adaptive gene duplications.

Despite their potential shortcomings, estimates of adaptive fixation rates derived from the MK framework do reflect the underlying biology (e.g., Huber et al. 2017). For example, theory predicts that selective interference among linked loci will impede adaptive fixation (Hill and Robertson 1968; Charlesworth 2012), and the MK framework does estimate lower rates of adaptive protein evolution for genes in regions with low or no recombination (e.g., Jackson et al. 2015). In *Drosophila*, it has been suggested that this interference reduces the impact of positive selection by nearly 30% (Castellano et al. 2016). Estimates of adaptive fixation also vary with genomic context and the level and pattern of gene expression, such that genes on the X- (or Z-) chromosomes often show higher rates of adaptive evolution than autosomal genes (reviewed in Meisel and Connallon [2013]). Finally, and perhaps most compellingly, it is precisely those protein-coding genes that we expect to display high rates of adaptive fixation a priori, such as genes potentially engaged in resistance to parasites and pathogens, that give the highest estimated rates (e.g., Haerty et al. 2007; Obbard et al. 2009; Enard et al. 2016; Castellano et al. 2019).

Such contrasting comparisons between gene classes provide a powerful way to gain a deeper understanding into rates of adaptive fixation. For example, house-keeping genes are expected to show lower rates of adaptive fixation than nonhouse-keeping genes (Hurst and Smith 1999), whereas genes involved in coevolutionary arms races are notorious for their accelerated rates of adaptive fixation. For example, male–female conflict over optimal investment and male–male competition to fertilize eggs are thought to have led to the high rates of evolution seen in the gamete recognition and fertilization proteins of many species and in some of the accessory proteins of *Drosophila* (Begun et al. 2000; Swanson et al. 2003; Kern et al. 2004; Pröschel et al. 2006a; Haerty et al. 2007; Vacquier and Swanson 2011). Conflict between genomic components that can “cheat” Mendelian inheritance, either through biased transmission (such as segregation and sex-ratio distorters) or through over-replication (such as endogenous retroviruses and transposable elements) also appear to drive high rates of adaptive protein fixation (Presgraves 2007; Rowley et al. 2018). Notably, proteins that mediate heterochromatin formation have been implicated in the suppression of both classes of genetic parasites (Czech and Hannon 2016; Helleu et al. 2016), and show high rates of adaptive fixation in invertebrates (Blumenstiel et al. 2016; Palmer et al. 2018).The antagonistic interaction between hosts and parasites is thought to explain why genes related to immune function display significantly higher rates of adaptive protein fixation than other genes (Downing et al. 2009; Enard et al. 2016; Ebel et al. 2017). Some specific immune pathways—such as the IMD pathway of *Drosophila* and the antiviral RNAi pathways of insects and nematodes—show consistently high rates (Obbard et al. 2009; Palmer et al. 2018; but see Hill et al. 2019). These can be several-fold higher than the genome-wide average, suggesting that host–parasite interactions may drive a disproportionate high fraction of amino-acid fixations in species (Obbard et al. 2009; Enard et al. 2016).

To obtain a clearer picture of the association of adaptive fixation with protein function on the one side and genomic context on the other side, we need whole-genome data sets from species that not only differ in their life history and ecology but also have substantial molecular and genetic characterization. Suitable population-genomic data sets are typically drawn from medically important species, or from experimental models such as *Drosophila*, *Arabidopsis*, yeast, and mice (Carreto et al. 2008; Horton et al. 2012; Lack et al. 2015; et al. 2016; Lusis et al. 2016). However, the strong historical focus on laboratory models such as *Drosophila* may have led to a biased perspective. The freshwater planktonic crustacean *D. magna* instead offers an ideal combination of genomic characterization and ecological context, allowing us to test whether insights from population-genetics archetypes like *Drosophila* can be generalized. *Daphnia magna* has been the subject of sufficient transcriptomic and genetic analysis that we can place their molecular evolution into a genomic and functional framework (Colbourne et al. 2011; Ye et al. 2017). *Daphnia magna* is also well studied in terms of its ecology, particularly the ecology and evolution of potential “arms-race” traits such as sex (Daphnia are facultatively asexual; Decaestecker et al. 2009), predator/prey interactions (Roozen and Lürling 2001), and parasitism (Ebert 1995; 2005; Toenshoff et al. 2018). The interactions between *D. magna* and its bacterial parasite *Pasteuria ramosa* is one of the best studied examples of coevolution (Decaestecker et al. 2007; Bento et al. 2017, 2020).

Here, we present a genome-wide analysis of adaptive protein fixation in the cyclic parthenogen *D.magna*, based on high coverage genomic sequencing of 36 diploid clones sampled across the Western Palaearctic, and outgroup sequences from the closely related species *D. similis* and *D. lumholtzi*. In contrast to *Drosophila melanogaster* and many other small invertebrates with large effective population size, we find low rates of adaptive protein fixation that are statistically indistinguishable from zero. This did not change after attempting to account for weakly deleterious mutation by modeling the DFE in several different ways. The inferred DFE suggests that, relative to *Drosophila*, there are low levels of constraint acting on amino acid polymorphism in *D. magna*. Finally, genes that are only expressed in one sex—which show high levels of adaptive protein fixation in many taxa—show rates of adaptive fixation and levels of constraint significantly lower than the genomic background in *Daphnia*, an observation that is consistent with selective efficacy differing between sexes. Using forward genetic simulations, we explore possible reasons for the low estimated rates of adaptive evolution.

## Results and Discussion

### Genome Sequencing and Annotation

**Fig. 1.**
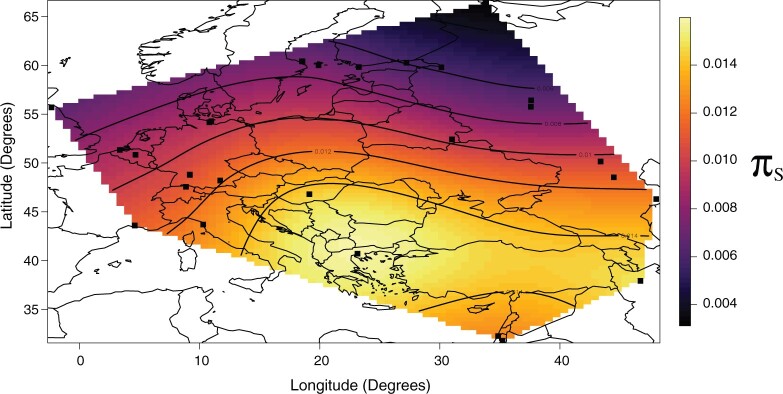
Synonymous genetic diversity ($\pi_{S}$) across the sampling range. $\pi_{S}$ has a mean value of 0.009 (range 0.002–0.021), with a clear pattern of decreasing genetic diversity in moving from Southeastern Europe and the Middle East to Northern and Western Europe.

We sequenced one diploid clone of *D.magna* from each of 36 locations in central and western Europe and the Middle East, and Eastern Asia (fig. 1) to high coverage (mean = ∼44X, std. dev. = 20.1) using the Illumina platform. Raw data are available under accession PRJNA480405. We mapped reads to the *D. magna* reference genome (daphmag2.4; GenBank GCA_001632505.1 PRJNA298946). To provide outgroup sequences, we additionally sequenced the genomes of *D. magna’*s close relatives, *D. similis*, and *D. lumholtzi* using Illumina short paired-end sequencing. Although not as contiguous as the *D. magna* genome, these outgroup sequences provided a comparable degree of biological completeness in coding sequences. To provide the primary outgroup sequence, we assembled a draft *D. similis* genome using MaSuRCA (Zimin et al. 2013) and identified the protein-coding sequences using both highly conserved BUSCO, related species, and species specific RNAseq data (BioProject accession number PRJNA533017). Using ORTHO-mcl (Li et al. 2003), we identified ∼12,000 putative 1:1 orthologs between *D. magna* and *D. similis* protein-coding genes. We aligned these as proteins, and excluded those with greater than 0.6 synonymous substitutions per site, which probably represent unidentified paralogs. After filtering, ∼11,000 coding sequences with a total length of ∼20 Mb remained. This represents approximately 42% of the protein-coding sequences originally annotated in *D. magna* 2.4 reference, suggesting either that a large proportion of *D. magna* proteins are recent in origin and so lack 1:1 orthologs with *D. similis*, or that the initial gene annotation of the *D. magna* genome was too permissive (Ye et al. 2017), or that the orthology detection approach we used has limited power in determining precise evolutionary relationships of a large number of highly similar genes of recent evolutionary origin. Molinier et al. (2018) identified a similar number of 1:1 orthologs when comparing de novo transcriptomes of *D. magna*, *D. pulex*, and *D. galeata*, showing that our observed counts are similar to other published studies on related systems. Finally, in comparing the *D. pulex-arenata* genome (clone TCO)—the first published genome in the genus—to the PA42 genome of *D. pulex*, two genomes that are much more closely related than *D. magna* and *D. similis*, (Ye et al. 2017) identified ∼12,000 1:1 orthologs. To provide a second outgroup to aid the polarization of *D. magna* polymorphisms, we additionally used a reference-assisted assembly of *D. lumholtzi*. This provided information for all of the focal protein-coding sites.

### *Daphnia magna* Effective Population Size and High Levels of Population Structure

**Fig. 2.**
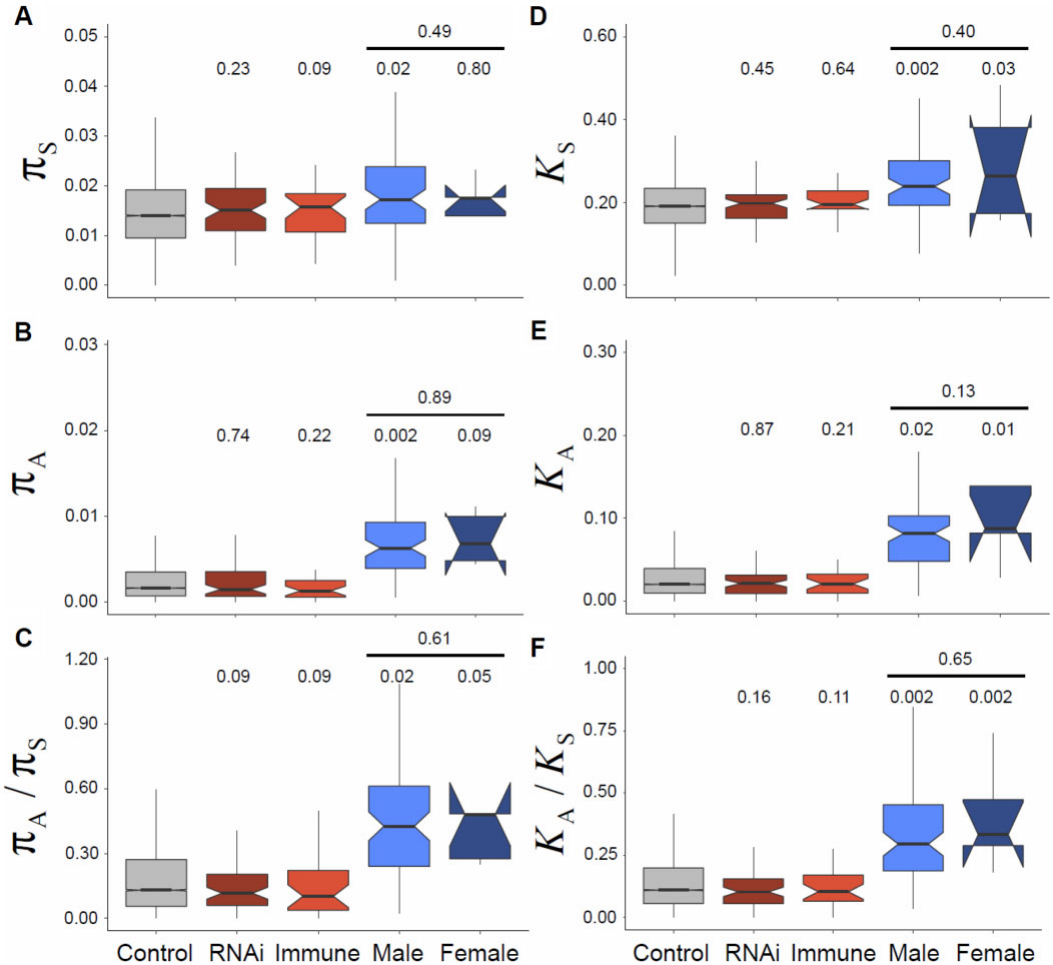
Diversity and divergence, as measured by *π* (left: *A*, *B*, *C*) and *K* (right: *D*, *E*, *F*), for synonymous sites (*π*_S_ and *K*_S_ in *A* and *D*), nonsynonymous sites (*π*_A_ and *K*_A_ in *B* and *E*), and the ratio of synonymous and nonsynonymous sites (*π*_A_/*π*_S_ and *K*_A_/*K*_S_ in *C* and *F*), respectively. Diversity and divergence measures are given for each gene class (see main text for explanation), including Control, which is an aggregate of all other genes not included in a subgene class. *P* values represent tests against “control” genes, with an additional test for the difference between male and female-biased genes. Note that the very small sample size leads to the confidence “notch” for the median of female-biased genes being wider than the inter-quartile range.

Synonymous variants experience relatively little constraint compared with nonsynonymous variants, so that their mean pairwise diversity within species (*π*_S_) can be treated as a proxy for unconstrained diversity. Overall synonymous diversity in our sample of European and Asian *D. magna* was *π*_S_ = 1.5% (consistent with a previous analysis on a smaller subset of genes by Haag et al. 2009), and mean within-population synonymous heterozygosity (based on a single diploid individual per pond) was 1.1% (fig. 2). This is lower than that of *D. melanogaster* in its native African range (*π*_S_ = 1.65%; e.g., Lack et al. 2015), and the North American daphniid, *D.pulex* (*π*_S_ =1.83%; Lynch et al. 2017). The de novo mutation rate for *D. magna* has very recently been estimated at 8.96 × 10^−9^ (CI: 6.66–11.97 × 10^−9^) mutations site^−1^ generation^−1^, but with a very wide range among clones spanning 3.57–33.53 × 10^−9^ (Ho et al. 2020). Assuming an island-model structured population, this corresponds to a global *N*_e_ in the range of 80–800 thousand (under such a model, local within-deme *π*_S_ is expected to be 4*Ndµ* where *d* in the number of demes and *N* the size of each deme; note that under such a model the within-population *π*_S_ is not affected by the degree of structure; Charlesworth and Charlesworth 2010, equation 7.4, p. 318). This is slightly lower than that of other small, highly abundant, and broadly distributed arthropods (Petit and Barbadilla 2009). However, diversity shows a clear South–North cline (fig. 1; see also Walser and Haag 2012): the highest diversity was observed in Southern Europe (Greece, *π*_S_ = 2.1%) and the lowest in Northern Europe (Finland, *π*_S_ = 0.37%). This variation is much greater than that seen in *D. melanogaster* over the same geographic range (*π*_all-sites_ in the range 0.50–0.62%; Kapun et al. 2020). This cline is consistent with three, nonexclusive, explanations. First, *D. magna* is thought to have undergone a large postglacial range expansion from the South-East of the European range, which could result in colonization bottlenecking and thus reduced diversity at the range margins (Fields et al. 2018). Second, regular local extinction/colonization dynamics in short-lived ponds, as they are common in the north of its range (“rockpools”), could also lead to recurrent bottlenecking, which would reduce diversity (Pannell and Charlesworth 2000; Haag et al. 2005). Third, *D. magna* is cyclically asexual, and very strong clonal dynamics in more ephemeral ponds may lead to high rates of drift and strong inbreeding and thus low heterozygosity (Ingvarsson 2002; Hartfield et al. 2016).

**Fig. 3.**
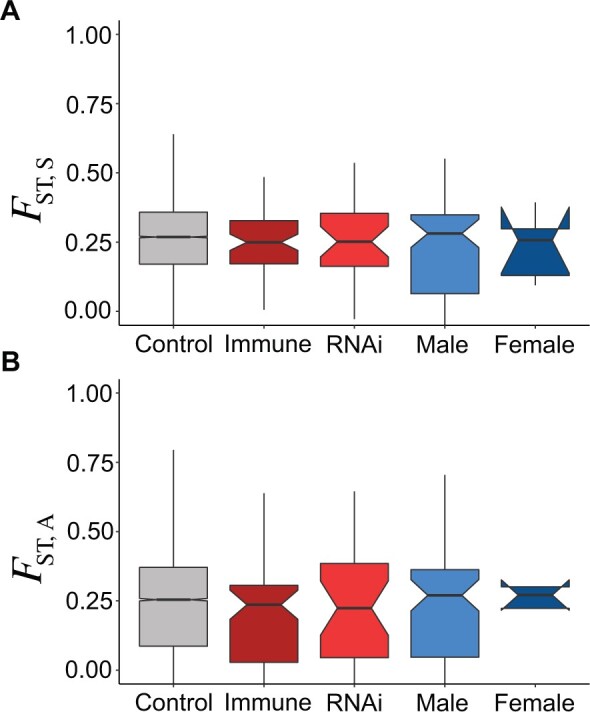
Mean population differentiation *F*_ST_, for (*A*) synonymous and (*B*) nonsynonymous sites across different gene classes. Note that the very small sample size leads to the confidence “notch” for the median of female-biased genes being wider than the inter-quartile range.

**Fig. 4.**
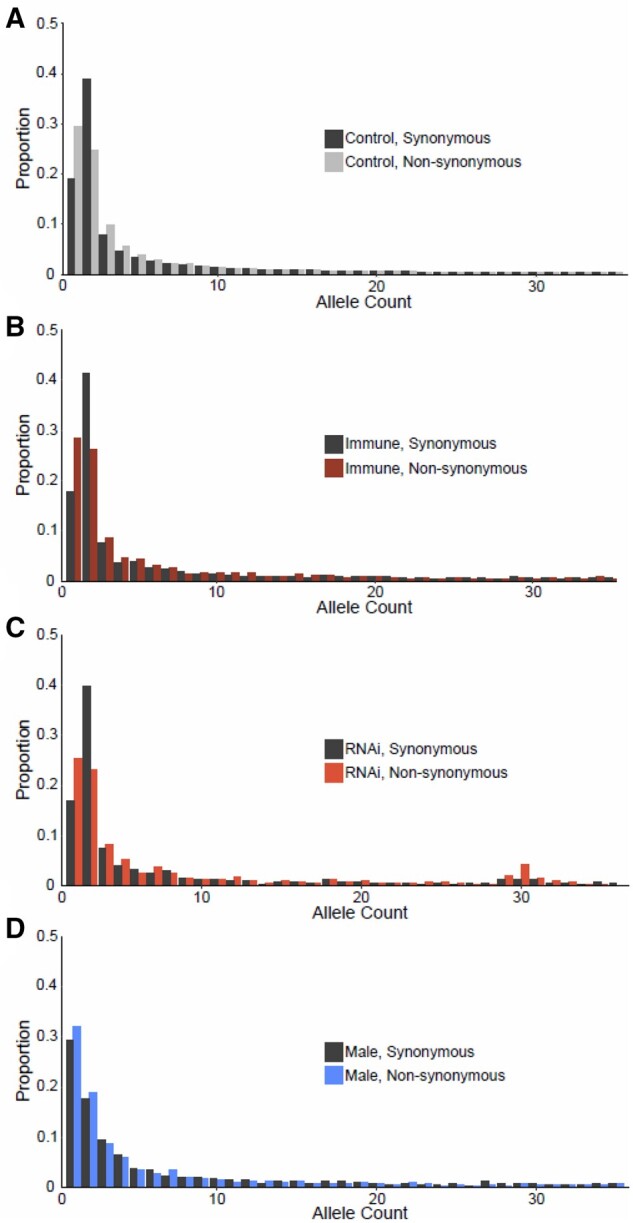
Folded SFS for (*A*) control (nonimmune, nonsex-biased), (*B*) immune, (*C*) RNAi, and (*D*) male-limited gene classes. We exclude the female-limited gene class due to the small number of genes leading to a very sparse SFS. Note that the excess of doubleton variants is consistent with the sampling of single diploid individuals from demes within a highly structured population, see supplementary figure 6, Supplementary Material online.

**Fig. 5.**
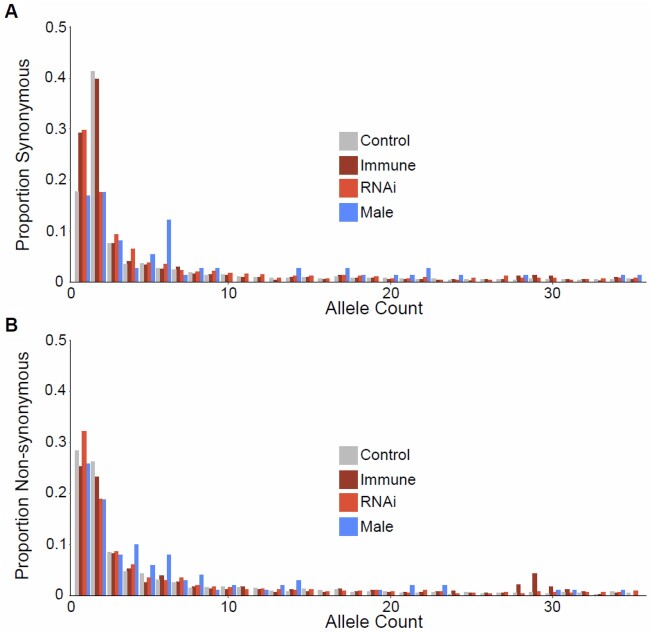
Folded SFS for all gene classes for (*A*) synonymous and (*B*) nonsynonymous sites, as a proportion of counts rather than raw counts. Note that the excess of doubleton variants is consistent with the sampling of single diploid individuals from demes within a highly structured population, see supplementary figure 6, Supplementary Material online.

Overall, genetic differentiation among subpopulations is often quantified using estimates of *F*_ST_, which can be considered as a deviation from Hardy–Weinberg equilibrium (HWE) caused by population subdivision (or as the variance in allele frequencies due to differences among populations). With a single diploid individual per location, it is not possible to separate *F*_ST_ from *F*_IS_ (the deviation from HWE caused by nonrandom mating within subpopulations). But, as most *Daphnia* populations are reported to be close to HWE following sexual reproduction (Haag et al. 2005; Walser and Haag 2012), *F*_IT_ is likely to be close to *F*_ST,_ and we refer to this statistic as *F*_ST_ below, though it could be an overestimate when rates of asexuality and thus intra-clone mating are high. Our data suggest that *D. magna* populations are substructured, with overall *F*_ST_ = 0.26 for synonymous sites (fig. 3). This observed level of population substructure is very high when compared with European *D. melanogaster*, for which *F*_ST_ across a similar geographic range is approximately 0.05 (Kapun et al. 2020). This pronounced substructure is consistent with the biology of *D. magna*, with its high variance in opportunities for dispersal between water bodies, persistent founder effects and potential for local extinction, strong clonal dynamics within ponds, and the combination of these dynamics with local adaptation (Walser and Haag 2012). Genetic structure is also clearly detectable in the overall folded site frequency spectrum (SFS), in which doubleton SNP numbers (those appearing twice in the data set) are substantially elevated (figs. 4 and 5). This doubleton excess reflects the relatively high rate of coalescence within ponds compared with migration between them (i.e., increases in *F*_ST_ correlate with more rapid coalescence between samples within populations compared with samples among populations; Whitlock 2011), that is, a low rate of dispersal (or an ancient timing of vicariance) among ponds, relative to local effective subpopulation size (see supplementary fig. 6, Supplementary Material online; Walser and Haag 2012; Fields et al. 2018).

### The *D. magna* Proteome Appears to Have a Relatively High Proportion of Weakly Deleterious Mutations

Selection on protein sequences alters nonsynonymous diversity (*π*_A_) and divergence (*K*_A_) relative to an unconstrained, neutral, expectation. In general, most amino-acid changing mutations are deleterious, reducing *π*_A_/*π*_S_ and *K*_A_/*K*_S_ ratios below one, and skewing the nonsynonymous SFS toward low-frequency variants. The impact of constraint is clearly evident in *D. magna* proteins, with overall mean *π*_A_/*π*_S_ = 0.24 and *K*_A_/*K*_S_ = 0.18 (fig. 2), which is similar to sexual *D. pulex* (documented *π*_A_/*π*_S_ = 0.24 and *K*_A_/*K*_S_*=* 0.25 [Tucker et al. 2013; Ye et al. 2017]). Although far below one—thus indicative of strong constraint—these ratios are similar to those seen in many large vertebrates and eusocial insects (Galtier 2016), but higher than that seen in *D. melanogaster* and many other arthropods (Chen et al. 2017), consistent with relatively low levels of constraint given the effective population size in *D. magna*.

**Fig. 6.**
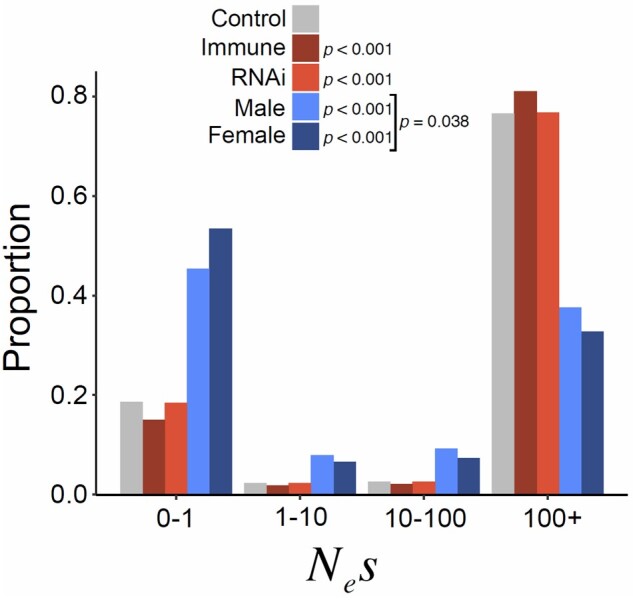
The estimated discretized DFE (distribution of deleterious fitness effects) across variants (Keightley and Eyre-Walker 2007; Eyre-Walker and Keightley 2009) for each gene class with the proportion of mutation with *N*_e_*s* values in each category. Both the control, and especially the male and female-biased gene classes, showed an upward bias for weakly deleterious mutations. Significant differences between individual DFEs were assessed using a likelihood-ratio test, wherein a model containing both control and class-specific genes as a single group and a model of each gene class having its own DFE were compared, with two degrees of freedom. A similar test was conducted between male and female-biased gene classes.

The skew in the SFS of nonsynonymous mutations (relative to the less constrained synonymous sites; figs. 4 and 5) can be used to estimate the DFE across mutations (Keightley and Eyre-Walker 2007; Eyre-Walker and Keightley 2009). The DFE is often parameterized as a gamma distribution and can be summarized as the proportion of mutations estimated to fall in different discrete categories of *N*_e_*s* (with *s* being the estimated selective disadvantage of the mutant). Using the program ‘DFE-alpha’ (Keightley and Eyre-Walker 2007; Eyre-Walker and Keightley 2009) to estimate the deleterious DFE, we found strongly deleterious mutations to be common in *D. magna*, with 77% in the category *N*_e_*s *=* *100+ (fig. 6). This is similar to several insect species for which large-scale population-genomic data are available, for example, 80–86% of variants in *D. melanogaster*, *Apis mellifera*, and *Anopheles gambiae* are similarly strongly deleterious (Palmer et al. 2018). However, *D. magna* displayed a larger estimated fraction of very weakly deleterious mutation with 19% having *N*_e_*s* < 1 as compared with only 3–6% in *Drosophila*, *Apis*, and *Anopheles*. This high proportion of very weakly deleterious mutations is consistent with the observation that *π*_A_/*π*_S_ > *K*_A_/*K*_S_, as weakly deleterious mutations are likely to persist on average in the population for longer periods than more strongly deleterious mutations, which are more quickly removed by selection. We also explored potential alternative distributions for the DFE using the program multiDFE (Kousathanas and Keightley 2013). Using MultiDFE, we identified two-step and three-step distributions to be a better fit, based on the Akaike Information Criterion (supplementary table 1, Supplementary Material online). However, the results were qualitatively similar, with 79% of mutations strongly constrained (*N*_e_*s* > 100) and 21% in the range 0.1 < *N*_e_*s* < 1, and the models differed little when characterized in four discrete categories.

### The *D. magna* Proteome Shows Low Levels of Global Adaptive Fixations

Under the MK framework (reviewed in Booker et al. [2017]) the amount of adaptive global fixation at potentially selected sites (here taken to be nonsynonymous) can be estimated from the ‘excess’ substitution over that predicted under the assumption that segregating amino-acid polymorphisms and all synonymous mutations behave effectively neutrally, that is, an excess of *K*_A_/*K*_S_ over *π*_A_/*π*_S_. In this context, a ‘fixation’ corresponds to a difference between species that is not polymorphic within species. This excess fixation is most often quantified as *α*, the proportion of amino acid substitutions that are adaptive, or *ω*_a_, the number of adaptive amino acid substitutions per site, normalized by the putatively unconstrained synonymous substitutions per site, that is, *K*_adaptive_/*K*_S_ (Booker et al. 2017). A traditional single-gene MK analysis of 10,687 *D. magna* genes identified 4,360 genes with significantly positive estimates of α (supplementary table 3, Supplementary Material online), but only 247 of these remain significant at a false-discovery rate (FDR) of 0.05. Among these, notable cases include genes with known, putative functions (Voltage-dependent L-type calcium channel subunit alpha, Calponin-homology [CH] domain-containing protein—actin binding, pre-mRNA-processing factor 19, and Histone chaperone ASF1A) but also uncharacterized proteins. However, for our data overall, the excess of *π*_A_/*π*_S_ over *K*_A_/*K*_S_ necessarily gives simple estimates that are negative. This was true both when summing polymorphism and divergence across all genes using the naïve estimator *D*_N_ – (*D*_S_*P_N_*)/*P*_S_ (*α* = −0.08, *ω*_a_ = −0.018) and when using maximum likelihood to fit a single *α* parameter, but with gene-specific diversity and constraint (*α* = −0.15; supplementary table 1, Supplementary Material online; Welch 2006).

Negative estimates of *α* are generally thought to reflect the downward bias introduced by the presence of weakly deleterious amino acid variants. Methods that account for this bias by excluding rare variants (Charlesworth and Eyre-Walker 2008) or by explicitly modeling the DFE, generally obtain positive and/or higher estimates (Booker et al. 2017). However, for *D. magna* we found that, although estimates were higher when we explicitly modeled the DFE (described above; *α* = −0.009, 95% CI −0.014 to −0.004), they were still not significantly greater than zero (fig. 8). Our estimate of *α* was similar regardless of whether the DFE was assumed to follow a gamma, log-normal, spike, or step distribution (supplementary table 2, Supplementary Material online). It was also true for the asymptotic estimate of *α* (supplementary fig. 1, Supplementary Material online), which generalizes the exclusion of rare alleles by considering the asymptotic estimate of *α* with increasing allele frequencies (Haller and Messer 2017a). For DFE-alpha, these findings were also robust to the use of all data (both alleles per population, including all populations) or subsampled data (one allele per population resulted in an increase in the estimated $\omega_{a}$ of 0.01, or excluding divergent Asian sample resulted in an increase in the estimated $\omega_{a}$ of 0.0006, for our control gene set). For Multi-DFE and asymptotic-MK, they were robust to the use of one or two outgroups to infer ancestral state for the unfolded SFS (supplementary fig. 1, Supplementary Material online).

These results appear to suggest that across the entire genome, the amount of global adaptive fixation in protein-coding sequences of *D. magna* is indistinguishable from zero, which contrasts sharply with many other small arthropods that have similar levels of synonymous diversity (e.g., Galtier et al. 2018). Given *Daphnia*’s cyclical asexuality and high levels of population structure, it is tempting to speculate that this may partly reflect the reduced efficacy of selection associated with increased selective interference (Hill and Robertson 1968; Comeron et al. 2008), particularly in the most extreme form of clonal interference (Neher 2013). However, estimates may also be downwardly biased by the presence of locally adapted nonsynonymous variants, as described above in the context of the DFE. Local adaptation is likely to be a widespread (Hereford 2009) and this is perhaps the most likely explanation of the deceptively limited signal of adaptive substitution here. Indeed, a recent study by Lynch et al. (2017) has shown that, within a single large population of *D. pulex*, positive selection can be efficient. Numerous studies in *D. magna* have suggested an important role of local adaptation in driving species-wide patterns of genetic diversity across a number of genes of ecological and evolutionary importance (Weider and Hebert 1987; Teschner 1995; Cousyn et al. 2001; Fisk et al. 2007; Allen et al. 2010; Agra et al. 2011; Miner and Kerr 2011; Roulin et al. 2013; Yampolsky et al. 2014; Radzikowski et al. 2018; Seefeldt and Ebert 2019). In principle, such local adaptation might be detectable in the MK framework, by considering differences among populations or regions as “fixed differences.” However, the timescales over which such local “fixations” could occur in *Daphnia* (tens of thousands of years) are so short compared with the divergence among species (millions of years), that they are unlikely to be detectable.

Finally, it is also possible that estimates are downwardly biased by the presence of “balanced” nonsynonymous alleles, which are maintained as polymorphisms for an extended period by negative frequency dependent selection (thus increasing *π*_A_) and are less likely to fix. Although such alleles, whereas they probably exist in all species and are implicated in the well-studied *Daphnia*–*Pasteuria* coevolutionary interaction (Routtu and Ebert 2015; Bento et al. 2017; Ameline et al. 2021; Bento et al. 2020), they only represent a small fraction of all genes (Andrés et al. 2009; Roux et al. 2013; Fijarczyk and Babik 2015; Croze et al. 2017) and are unlikely to have a large impact on genome-wide estimates.

### Biased Estimates of the DFE and *α* Caused by Population Structure Alone Are Unlikely to Explain These Results

Local adaptation and/or the reduced efficacy of selection due to population structure and clonal interference are likely explanations for our low estimate of *α*, but it is also possible that the demographic history and/or our sampling strategy have led to biased estimates. DFE-alpha (Keightley and Eyre-Walker 2007; Eyre-Walker and Keightley 2009) and Multi-DFE (Kousathanas and Keightley 2013) do attempt to account for deviations from a standard neutral SFS caused by population size changes by modeling a step-change in size. Simulations have shown that this makes their inferences surprisingly robust to a more complex history of population sizes, enabling them to accurately recover alpha and the form and parameters of the DFE from simulated data (Keightley and Eyre-Walker 2007; Eyre-Walker and Keightley 2009; Kousathanas and Keightley 2013). However, our data differ from previous work in two ways. First, the *D. magna* population is substantially more structured than many other species (overall *F*_ST_ =0.26 in our data, above), and second, our sampling strategy used a single diploid individual from each of many distinct demes. To assess the likely impact of these factors, even in the absence of local adaptation, we used forward simulation with SLiM 2.0 (Haller and Messer 2017b) to examine the performance of DFE-alpha, Multi-DFE, and asymptotic-MK on data sampled from a finite island population of 36 demes. We chose natural selection parameters to match those inferred for *D. melanogaster* (with which we wished to contrast our *Daphnia* estimates), but (scaled) mutation and recombination parameters to mimic our *D. magna* estimates, with low, medium, and high rates of migration (*F*_ST_ = 0.76, 0.26, 0.05, respectively) and low, medium, and high rates of sexual reproduction (every generation, every 8^th^, and every 80th generation, in addition to a low background rate of 1% sexual individuals in an asexual generation). We also examined the impact of three different sampling strategies: 1) one diploid per deme (as in our data), 2) an equal-effort approach of 36 diploids from a single deme (as done in most similar studies), and 3) a higher-effort approach of 360 individuals spread evenly across demes.

**Fig. 7.**
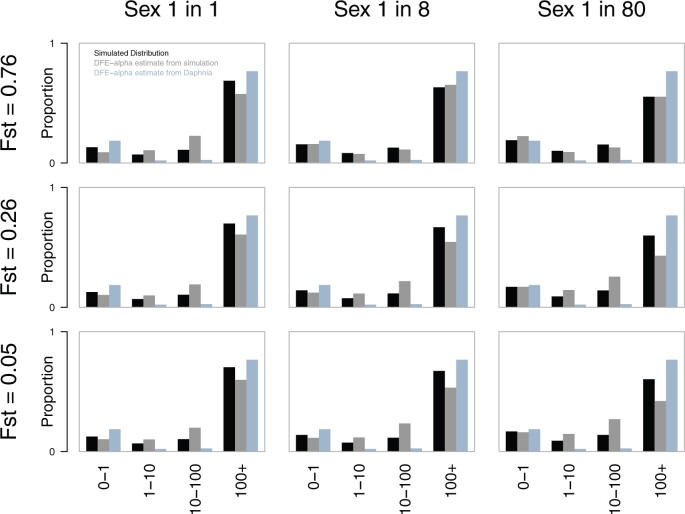
Simulated (black) and estimated (gray, as shown for the controls in fig. 6) DFE proportions for three levels of frequency of sexual reproduction and population subdivision (*F*_ST_), respectively. Using SLiM 2.0, we simulated coding DNA sequence-like data for one diploid individual sampled from each deme of a 36-deme structured population and inferred the DFE and α. Mutation and recombination rates were selected to match those of *D. magna* (after scaling, see Materials and Methods), and selection coefficients were chosen to reflect those estimated for *D. melanogaster*, which generally shows a high proportion of adaptive substitutions and a small proportion of weakly deleterious mutations. Combinations of population subdivision (and thus migration) and sexual reproduction rates were explored, bracketing credible values for *D. magna*. Consistent with theoretical expectations, strong population subdivision (low migration) reduced the efficacy of natural selection, and rare sex reduced the efficacy of positive selection, at least in part through the constraint which arises from link sites. The estimated proportions of DFE were broadly consistent with estimates of α observed in the simulation (table 1; see supplementary table 3, Supplementary Material online for observed values). These results suggest the effects of population structuring and asexuality (which are unaccounted for by DFE-alpha or asymptotic MK) are in the general direction of but still insufficient to completely obscure *Drosophila*-like levels of adaptive protein substation.

We found that, despite the extreme mismatch between the population structure we simulated and the one we fitted, we found a surprisingly good match between the true (simulated) parameters and those inferred by DFE-alpha, at least for intermediate and low levels of population structure and asexual reproduction (fig. 7 and supplementary figs. 6–8, Supplementary Material online). DFE-alpha, which assumes a gamma-distributed DFE (as was simulated), did appear to slightly over-estimate alpha. However, the effect was generally quite small (supplementary fig. 7, Supplementary Material online). Multi-DFE performed similarly, albeit with a greater degree of bias, but was consistently misled as to the form of the DFE, preferring (by AIC) the step and spike distributions over a gamma distribution. In contrast, asymptotic-MK generally slightly underestimated *α*. Importantly, because none of the three methods gave zero or negative estimates with this sampling strategy, and the model-based methods generally gave over-estimates of the proportion of adaptive substitution (*α*), our unusually low estimates of alpha cannot easily be ascribed merely to the impacts of population structure and structured sampling (supplementary fig. 7, Supplementary Material online).

Although we sampled one diploid individual per population, most similar studies took multiple individuals from a single local population. We were therefore interested to see whether the MK-framework approaches were similarly misled by the presence of population structure when sampling all individuals from the same deme. We found that this, standard, approach was no better than sampling one individual per deme, and may in fact be slightly worse in terms of the accuracy of the DFE—especially for very high levels of population structure and asexuality (supplementary fig. 7, Supplementary Material online). The proportion of adaptive substitutions was generally under-estimated, especially when structure was high and asexual reproduction dominant (supplementary fig. 8, Supplementary Material online), and Multi-DFE consistently mis-inferred the form of the DFE, preferring spike and step distributions over the simulated gamma distribution. Even sampling 10-fold more individuals, evenly spread across the structured population, did not substantially improve the outcome over sampling one individual per deme (supplementary fig. 9, Supplementary Material online). Although our simulations were limited in scope and more replication is required, they suggest that further investigation of the sensitivity of these methods to SFS deviations introduced by population structure may be warranted.

### Little Evidence for “Arms Race” Evolution and Very Low Constraint in Sex-Biased Genes

Although the overall evidence for adaptive protein evolution in *D. magna* is limited, genes that are widely presumed to be engaged in evolutionary “arms race” conflicts often display higher rates of adaptive protein evolution that the genome-wide average. For example, a previous analysis of *D. pulex* that compared 27 putative immune-pathway genes with 20 other genes reported a significantly higher *α* in the immune genes than the nonimmune genes *α*  =  0.33 versus −0.27, *P *=* *0.049 (McTaggart et al. 2012). We tested for an elevated rate of adaptive protein evolution in potential “arms race” gene classes, including 69 putative immune-related genes, 30 antiviral RNAi and piRNA genes, and 78 genes that show an strong male-bias in their expression. We did this in three ways. First, we used a maximum-likelihood implementation of the multigene MK test to estimate overall *α* and *α* for each class of gene function (Welch 2006). Second, to mitigate the impact of weakly deleterious variants, but conditional on a shared demographic history for the different gene classes, we used the software DFE-alpha (Keightley and Eyre-Walker 2007; Eyre-Walker and Keightley 2009) to infer the DFE, *α*, and *ω*_a_, for each gene class and tested among them using likelihood ratio tests and permutation tests. Note that, in light of our simulations (above), we chose to limit our analysis to the more widely used DFE-alpha rather than to explore a wider range of functional forms for the DFE. Third, we used a SnIPRe-like analysis, which recasts the MK test as a generalized linear mixed model (Eilertson et al. 2012; Palmer et al. 2018), to provide a more formal statistical test of the differences in selection between gene classes.

Surprisingly, despite our expectation that “arms race” genes would show a different level of constraint, the *π*_A_/*π*_S_ and *K*_A_/*K*_S_ ratios for immune and RNAi genes were not significantly different from those of the genome-wide background (fig. 2). Similarly, the gamma-distributed SFS did not differ substantially between these gene classes, suggesting only a small—albeit nominally significant—difference in the DFE (fig. 6). This resembles what is seen for RNAi genes in *D. melanogaster*, *A.mellifera*, and *A.gambiae*, where the DFE of RNAi-pathway genes is also slightly, but significantly, different to the genome-wide background (Palmer et al. 2018).

**Fig. 8.**
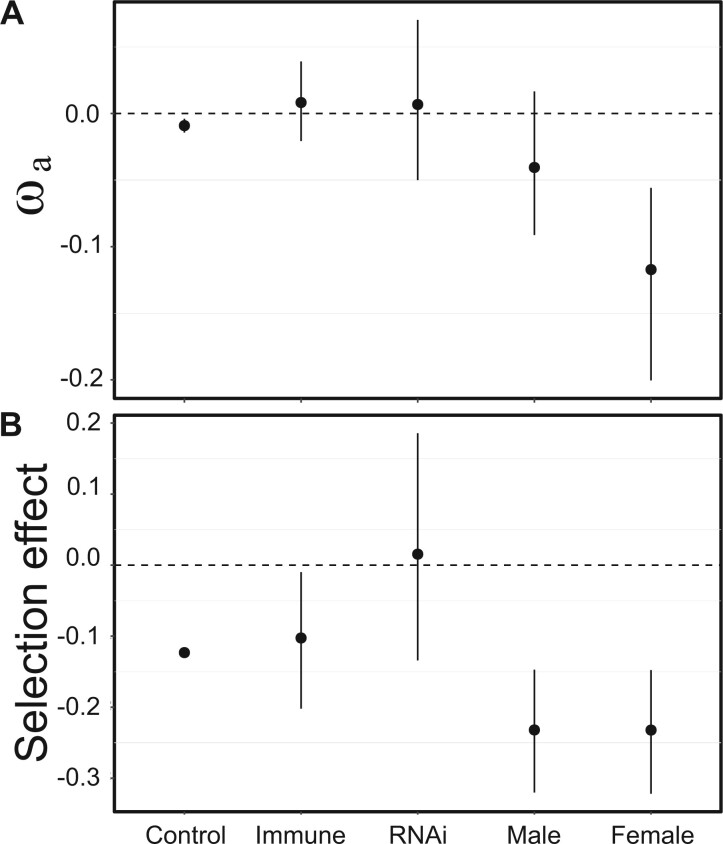
(*A*) $\omega_{a}$ (estimated with DFE alpha) estimates for each gene class including 95% bootstrap confidence intervals and (*B*) the posterior distribution of selection effects as estimated with our SnIPRe-like analysis associated with each gene type.

When ignoring the impact of weakly deleterious variants, maximum-likelihood estimates of the rate of adaptive amino-acid fixation did vary among the gene classes (ΔAIC = 115 between a single-*α* and five-*α* model; supplementary table 2, Supplementary Material online), but bootstrap intervals for *α* overlapped zero for immune (*α* = −0.00 [−0.34, 0.24]), RNAi (*α*  = 0.08 [−0.06, 0.18]), male (*α* = −0.08 [−0.41, 0.15]) and female biased (*α*  = 0.07 [−0.64, 0.35]) gene classes, whereas *α* for other genes (“control”: *α* = −0.16 [−0.13, −0.19]) genes was significantly negative. The SnIPRe-like analysis gave similar results, with strongly negative genome-wide and immune-pathway estimates of the selective effect, but with substantially higher and marginally significant estimates for RNAi genes (fig. 8*B*; mean selection effect = 0.015; *P* < 0.05). Explicitly modeling the DFE in *D. magna* did not lead to significantly higher or positive estimates of *α* or *ω*_a_ for immunity genes than other genes (fig. 8*A*; although the RNAi-pathway genes were substantially, but not significantly, higher, $\omega_{a}=0.0067,\;P>0.05)$. This is in contrast to the impact of modeling the DFE in MK-like analyses of *D. melanogaster*, *A.mellifera*, and *A.gambiae*, where accounting for the presence of weakly deleterious mutations leads to positive estimates of *α*. Together, these results suggest that the rate of adaptive protein fixation occurring in *D. magna* immune-related proteins is not substantially different to that occurring in the proteome as a whole, and could again reflect an impact of cyclical asexuality and high population structure on the efficacy of selection. As neither *π* nor *F*_ST_, at either synonymous or nonsynonymous sites, was significantly elevated in RNAi or immune-pathway genes (figs. 2 and 3) this is not easily attributable to generally increased balancing selection or local adaptation obscuring elevated adaptive substitution in these gene classes relative to the genome average.

Unlike the immune and RNAi-pathway genes, *π*_A_/*π*_S_ and *K*_A_/*K*_S_ ratios of male-biased genes were both significantly elevated compared with the genomic background (0.52 and 0.37 respectively; fig. 2). This was matched by an extremely large increase in the estimated proportion of very weakly deleterious mutations (45% 0.1 < *N*_e_*s* < 1; fig. 6) and a concomitant decrease in strongly deleterious mutations (35% *N*_e_*s* >100). It also corresponded to more negative estimates of *α* and *ω*_a_, and the SnIPRe-like selective effect (p≪0.01). Together, these results suggest that the most strongly male-biased genes of *D. magna* show low levels of constraint, and likely low rates of adaptive nonsynonymous substitution. This contrasts sharply with the higher estimates of *α* seen for male-specific genes in other taxa (Pröschel et al. 2006) and could perhaps suggest that the relative infrequency of sexual reproduction in *D. magna* has reduced the strength of selection acting on male-specific genes. However, an equivalent analysis of five genes expressed in asexual females but not in males gave quantitatively very similar results, showing high levels of nonsynonymous polymorphism and a high proportion of very weakly deleterious alleles. Although the small number of genes meant that the power of this analysis was low, it could suggest that the particularly low constraint in male/female-specific genes relative to the genome as a whole was not attributable to their role in sexual reproduction given that female-specific genes may be under selection even if sexual reproduction is not occurring every generation.

## Conclusions

We studied samples of *D. magna* that covered about a third of this species' Holarctic distribution (we did not include East Asian and North American sites). Overall, we found that *D. magna* displayed relatively high synonymous site diversity, only slightly lower than that of other small invertebrates with large census population sizes, and consistent with a large coalescent effective population size. However, in contrast to *Drosophila*, diversity was very variable among populations and there was a high level of genetic structure, suggesting relatively low levels of dispersal. Although *Daphnia* is well known for local adaptation of diverse traits (Cousyn et al. 2001; Roulin et al. 2013; Reger et al. 2018; Seefeldt and Ebert 2019), we found little evidence for pervasive species-wide adaptive fixation in protein-coding genes, either in the genome as a whole or in putative “arms race” gene classes such as those involved in immunity, RNAi-based defence against viruses and transposable elements, and male-specific functions. This finding is in contrast to other arthropods such as *Drosophila* and *Apis*, as well as more broadly in other systems including humans and mice. Low rates of adaptive protein fixation were evident regardless of whether or not we attempted to account for the presence of weakly deleterious mutations. We speculate that this finding partly reflects an overall reduction in the efficacy of selection in the cyclical parthenogen *D. magna*, as might be expected from an increase in selective interference caused by this mode of reproduction and the highly structured geography of the populations. Furthermore, it is likely to be a consequence of pervasive local adaptation, which is well described for *Daphnia* in general and *D. magna* specifically (Cousyn et al. 2001; Roulin et al. 2013; Reger et al. 2018; Seefeldt and Ebert 2019), leading to downwardly biased estimates of species-wide adaptive fixation.

## Materials and Methods

### Samples and DNA Sequencing

We analyzed whole-genome sequences of 36 *D. magna* clones, 1 clone of *D. similis*, and 1 clone of *D. lumholtzi*. The *D. magna* genotypes (clones; *D. magna* can be maintained as stable, asexually propagated, genotypes) used in this study originated either from field collected plankton samples, were hatched from field-collected resting eggs, or resulted from inbred crosses in the laboratory (two clones). Field-collected planktonic females were brought to the laboratory, and individual females were allowed to reproduce asexually. Field-collected resting eggs (ephippia) were collected on the surface of pond sediments and were washed and stimulated to hatch by exposure to continuous light under room temperature in well-oxygenated medium. Hatchlings were isolated and clonal lines were produced and kept under conditions of continuous asexual reproduction. Two clones (*D. similis* and *D. lumholtzi*) were obtained by selfing of field-collected females. Selfing was achieved by allowing asexually produced sons to fertilize sexual eggs of their clonal sisters. The *D. similis* clone (from Israel) is the result of three rounds of selfing, the *D. lumholtzi* clone (from Zimbabwe, Africa) resulted from three rounds of selfing.

To reduce nonfocal DNA in our sequencing libraries (from microbiota and food items), individuals were treated for 72 h with three antibiotics (streptomycin, tetracycline, ampicillin) at a concentration of 50 mg/l each. Antibiotics were refreshed every 24 h. Clones were fed with dextran beads (Sephadex “Small” by Sigma Aldrich: 50 μm diameter) at a concentration of 0.5 g/100 ml to aid gut evacuation (Dukić et al. 2016). Animals were moved out of antibiotics and into 1.5-ml Eppendorf microcentrifuge tubes and excess fluids removed with a sterile pipette. Extraction buffer (Qiagen GenePure DNA Isolation Kit) was subsequently added to the tubes and tissue was disrupted using sterile and DNA-free plastic pestle. The resultant solution was incubated overnight with Proteinase K at 55 °C. RNA was degraded using RNAse treatment for 1 h at 37 ° C. Protein removal and DNA precipitation, including the addition of glycogen (Qiagen) to aid DNA precipitation, were done using the Qiagen GenePure DNA Isolation Kit instructions. Resultant purified DNA was suspended in 40 μl of Qiagen DNA hydration solution and subsequently tested for purity and concentration using a Nanodrop and Qubit 2.0, respectively. Libraries were either prepared using Kapa PCR-free kits and sequenced by the Quantitative Genomics Facility service platform at the Department of Biosystem Science and Engineering (D-BSSE, ETH), in Basel, Switzerland, on an Illumina HiSeq 2000, or were provided to Edinburgh Genomics (NCBI BioProject number PRJNA480405) for library preparation using TruSeq DNA Nano gel free kits and paired-end 125 nt sequencing using HiSeq v.4.

Read quality was assessed using FastQC v.0.10.1 (http://www.bioinformatics.babraham.ac.uk/projects/fastqc; released on March 5, 2012). Paired-end sequences were adapter trimmed and quality filtered using Trimmomatic v.0.36 (Bolger et al. 2014). After trimming of adapter sequences, terminal bases with a quality score below three were removed from both ends of each read. Then, using the sliding window function and again moving in from both sides, further 4 bp fragments were removed as long as their average quality score was below 15. Read quality was rechecked with FastQC to confirm quality and adapter trimmed succeeded. These high-quality reads were mapped to the *D. magna* reference genome (NCBI database; Assembly name: daphmag2.4; GenBank assembly accession: GCA_001632505.1, Bioprojects accession: PRJNA298946), consisting of 28,801 scaffolds, 38,559 contigs and a total sequence length of 129,543,483 bp) using BWA MEM (Li and Durbin 2009; Li 2013), the resulting sam alignment file being subsequently converted to a bam, coordinate sorted, and filtered for mapping quality ≥20 using SAMtools (Li et al. 2009).

To identify SNP polymorphisms we applied GATK v.3.8 (McKenna et al. 2010) HaplotypeCaller according to GATK Best Practices recommendations (DePristo et al. 2011; Van der Auwera et al. 2013), including a QD (quality by depth) ≥6 and a GQ (genotype quality) ≥20. We then used *vcflib* (https://github.com/vcflib/vcflib), specifically the *vcfgeno2haplo* module, to generate a revised version of the *D. magna* reference genome that includes the identified polymorphisms for individual clones. For each of these individual’s updated references, we extracted coding sequences using *gffread* from the gffcompare package (https://github.com/gpertea/gffcompare). We note that the large number of recent paralogs within the *D. magna* genome (Orsini et al. 2016) could lead to high rates of cross-mapping. However, because these reads will have low mapping quality, the affected genes will be excluded from our analysis.

### *Daphnia similis* Assembly

We used the MaSuRCA assembler (Zimin et al. 2013) to assemble the nuclear genome of the *D. similis* clone. The Illumina paired-end reads were used as input for MaSuRCA and were assembled into super-reads. The assembly procedure used default settings but varying the kmer size over larger ranges (21, 31, 41, 51, 61, 71, 81, and 91) and subsequently over a smaller, more targeted kmer range (63, 65, 67, and 69). The resulting assembly showing the lowest number of scaffolds was considered the most reliable for our purpose and used for further analyses. An assessment of the completeness of the newly assembled nuclear genomes was performed using BUSCOv3 (Waterhouse et al. 2018). A total of 1,066 single-copy arthropod genes were searched against our de novo genome assemblies. Annotation was made using the MAKER 2 (Holt and Yandell 2011 pipeline, which included the eukaryotic gene predictors GeneMark-ES 4.33 [Ter-Hovhannisyan et al. 2008]), Prodigal 2.6.3 (Hyatt et al. 2010), and Augustus 3.2.3 (Stanke et al. 2008). We used protein hints derived from the *D. magna* genome as well as paired-end, Illumina sequenced RNAseq data derived from the same *D. similis* clone (NCBI BioProject PRJNA744861).

### *Daphnia lumholtzi* Assembly

A single, short-insert PE library was generated for a second outgroup, *D. lumholtzi* (NCBI BioProject PRJNA744886)*.* Because a high-quality assembly is unlikely to result from such a data set, we applied a reference assisted assembly approach in order to provide additional context for polarizing variants. Specifically, as with reads sampled directly from *D. magna*, reads from *D. lumholtzi* were aligned to the *D. magna* reference and the same procedures were used to generate variant calls using the GATK variant caller. Next, we used the GATK FastaAlternateReferenceMaker approach in order to replace variants ascertained from *D. lumholtzi* in the *D. magna* reference genome.

### Orthology

The MK approach to inferring adaptive fixations requires divergence between two sequences, limiting the analysis to those genes with 1:1 homologs. Although it is, in principle, possible to instead use pairs of paralogs rather than orthologs, such analyses are prone to mis-inference as they violate one of the central assumptions: that constraint is thought constant over the interval that gave rise to both polymorphism and divergence data (Hahn 2009). Therefore, to identify 1:1 orthologs for analysis, protein sequences of *D. magna* (v2.4 GenBank: LRGB00000000), and *D. similis* genomes were used as inputs for OrthoMCL (Li et al. 2003), a fast method for inferring orthologous groups from protein sequences. For further analysis, we retained only those genes that were identified by OrthoMCL as single copy, one-to-one orthologs in both species. Following alignment (see more details below) we set a threshold of *K*_S_≤ 0.6 (based upon visual inspection of the full distribution of *K*_S_ values) to consider homologs as orthologs, rather than likely paralogs.

### Alignment

Alignments of orthologous coding sequences of *D. magna* and *D. similis* were made using a custom R script which would initially utilize the R package *seqinr* (Charif and Lobry 2007) to import individual coding sequences for each respective species, followed by identification of the correct reading frame, and finally a codon based alignment using PRANK (Löytynoja and Goldman 2005, 2008). To assess the quality of the alignment procedure, we calculated gene-wise *K*_A_, *K*_S_, and *K*_A_/*K*_S_ using the codeml function from the package PAML (Yang 1997, 2007). To further improve the quality of aligned coding sequences we excluded regions that contained stop codons, and very poorly aligned regions. Poorly aligned regions were identified as those with multiple consecutive codons possessing no aligned bases, and regions in which *K* or *K*_A_ was > 0.5. These parameters were selected after manually examining the impact of alternative masking strategies on the most divergent genes and those with the highest *K*_A_/*K*_S_ ratios to confirm that, even in the most divergent proteins, only poorly aligned regions were masked. Gene sequences deriving from the above variant calling approach were added into the *D. magna* and *D. similis* reference alignments using the MAFFT (Katoh et al. 2002; Katoh and Standley 2013) –add function, which aligns sequences to a previously generated multiple sequence alignment.

### Focal Gene Classes

We defined focal gene classes based on orthology and expression patterns. To identify orthologous sequences of well-characterized immune genes from a broad range of systems, we used an additional run of OrthoMCL which included protein sequences derived from *D.melanogaster*, *Caenorhabditis elegans*, and *Homo sapiens*. To identify male and female specific genes, we used RNAseq data derived from the study of Molinier et al. (2018; NCBI SRA PRJNA533017).

Briefly, the data set of (Molinier et al. 2018) is composed of high-coverage paired-end 100 bp Illumina reads derived from our different genotypes (clones) as biological replicates (Moscow, Russia, 55.763514, 37.581667). One library was prepared per genotype and sex, resulting in a total of eight libraries. Quality and adapter trimming procedures were the same as used above for whole-genome DNA sequencing. Reads were mapped to the same *D. magna* reference assembly as above (NCBI database; Assembly name: daphmag2.4; GenBank assembly accession: GCA_001632505.1, Bioprojects accession: PRJNA298946), but included this time the associated annotation file (in GFF) format, as well as a genome annotation with 26,646 genes, using the RNA-Seq aligner STAR (Dobin et al. 2013). The raw counts (number of mapped reads per transcript per sample) were obtained with the software featureCounts (Liao et al. 2014). We analyzed differential gene expression using DESeq2 (version 1.10.1) implemented in R (Love et al. 2014). Raw read counts were used as input data, and the subsequent analyses used the normalizations of read counts as performed by DESeq2. The male versus female comparison was carried out with a two-factor design taking into account clone identity and sex. We defined male and female-specific genes as those that displayed zero or nearly zero expression in the nonfocal sex and at least a 5-fold greater expression in the other sex.

### Population Genetics

We used a modified set of python scripts (https://github.com/tatumdmortimer/popgen-stats), which relied primarily on the EggLib (De Mita and Siol 2012) python library to calculate gene-wise $\pi_{A}$ and $\pi_{S}$, as well as generate the MK tables ($P_{N}$,${\;P}_{S}$, $D_{N}$, and $D_{S}$) to be used in subsequent analysis. For our SNIPre like analysis, which requires counts of synonymous and nonsynonymous codon sites, we used a perl script from SNAP v.2.1.1 (Korber 2000) to calculate these values based on mutational opportunity using the Nei and Gojobori method (https://www.hiv.lanl.gov/content/sequence/SNAP/perlsnap.html). To derive an estimate of *F*_ST_ for synonymous and nonsynonymous sites ($F_{ST,s}$ and $F_{ST,N}$, respectively) we used $\pi_{A}$ and $\pi_{S}$ estimates, and calculated *F*_ST_ = $\overset{-}{(\pi_{Ij}-\pi_{Tj})/\pi_{Tj}}$, where *j* represents a separate calculation for both synonymous and nonsynonymous sites (Weir 1996, eqn. 5.3, p. 174).

To quantify both the genome wide and gene-class specific signal of adaptive protein evolution in *D. magna* we used a subset of the approach described in Palmer et al. (2018), relying principally on two approaches derived from the MK test (McDonald and Kreitman 1991). The underlying logic of the MK test remains consistent within these methods, where the polymorphism and divergence data from putatively neutral and potentially selected variants are used to infer an excess of nonsynonymous fixations that can be attributed to positive selection (Booker et al. 2017).

First we used the maximum-likelihood approach of Welch (2006), which does not take into account the likely presence of weakly deleterious segregating polymorphisms. Using per-gene counts of the numbers of fixed differences and polymorphisms, we fitted a range of models that either fix *α* at zero, allow a single value of *α*, or allow each gene class to have a different α. We also fitted models that allowed constraint (*f*, in the terminology of Welch 2006) and the population mutation rate (θ) to vary among genes or gene classes, and we selected among models using Akaike weights (supplementary table 1, Supplementary Material online). Despite the large number of parameters, we found that models allowing all genes to differ in *f* and *θ* were the best supported, and it is these that we report. For the two best-fitting models, we used 1,000 bootstrap analysis (resampling within gene classes) to provide 95% bootstrap intervals around the estimates.

Second, we used DFE-alpha which uses an explicit population-genetic model to estimate the number of adaptive nonsynonymous substitutions per site, whereas simultaneously accounting for changes in population size and the distribution of deleterious fitness effects (Keightley and Eyre-Walker 2007; Eyre-Walker and Keightley 2009). We used 1,000 boot-strap iterations to generate a confidence interval around the observed DFE for each gene class type. Significant differences in the DFE for each gene class were assessed by using a likelihood-ratio test with two degrees of freedom. To confirm that our findings were not wholly dependent on the gamma distributed DFE assumed by DFE-alpha, we also explored alternative parameterizations using multiDFE, which permitted models with lognormal, gamma, and beta, 1–6 spike (point density), and 1–5 step (multiple continuous uniform, with estimated boundaries and densities) DFE.

Third, we used AsymptoticMK (Keightley and Eyre-Walker 2007; Eyre-Walker and Keightley 2009), which attempts to mitigate the impact of weakly deleterious alleles by finding global *α* in the limit of increasing derived-allele frequency. This is done by fitting a saturating curve to *α* inferred from polymorphism pooled into 20 “slices” of the unfolded SFS with 5% boundaries. Given the small number of genes in the immune, RNAi, and sex-biased classes, we did not apply AsymptoticMK to any subsets of the data. To infer an unfolded SFS we polarized variants as ancestral versus derived using the approach employed by est-sfs (Keightley and Jackson 2018). We used both *D. similis* and *D. lumholtzi* as outgroups to identify if an allele was ancestral or derived.

Finally, we used an extension of the SnIPRE model (Eilertson et al. 2012), which re-frames the MK test as a linear model in which polymorphism and substitution counts are predicted by synonymous or nonsynonymous state (Palmer et al. 2018). We used the re-implementation of the SnIPRE model by Palmer (Palmer et al.), which utilizes the Bayesian generalized linear mixed modeling R package *MCMCglmm* (Hadfield 2010). Briefly, we modeled the number of mutation counts in each of four classes: synonymous polymorphism, nonsynonymous polymorphism, synonymous divergence, and nonsynonymous divergence. The fixed effects portion of the model included effects for the nonsynonymous state, the divergence state, and a nonsynonymous: divergence interaction, effectively capturing constraint, divergence time, and the excess contribution of nonsynonymous mutations to species divergence, respectively. These fixed effects were estimated separately for the genome-wide background, immune genes, RNAi genes, and male or female-specific genes. We also fitted a fixed effect for gene length, although this was close to 1, indicating similar mutation rates across gene lengths. Finally, we estimated gene-specific random deviations from each of the four mutation classes, assumed to come from a multivariate normal distribution with an unstructured covariance matrix. From this model, the genome-wide “selection effect” is the nonsynonymous: divergence effect, and selection effects for specific gene groups (e.g., RNAi, sex-specific) are obtained by adding the genome-wide nonsynonymous: divergence effect to the nonsynonymous: divergence effect for the group in question. To test whether the selection effects of specific gene group significantly differed from the genome-wide average, we determined the proportion of the posterior distribution that overlapped zero for each of the gene group-specific nonsynonymous: divergence effects.

### SLiM Simulation

To explore the behavior of DFE-alpha and AsymptoticMK when used to analyze data drawn from a structured population, we used the forward genetic simulator SLiM 2.0 (Haller and Messer 2017b). We simulated a 500-kb chromosome encoding ten 5-kb “coding” loci separated by 5 kb “noncoding” regions, evolving in a finite island model (symmetric migration) with 36-demes of 500 diploid individuals for 1 million generations. Within coding regions, 24% of mutations were unconstrained (representing “synonymous” mutations) and 76% were potentially selected. The mutation rate and recombination rate were constant across the “chromosome,” with scaled values chosen to reflect empirical values (*µ* = 5.6 × 10^−9^, as estimated for *D. pulex* (Keith et al. 2016), and within the range recently reported across *D. magna* genotypes (Ho et al. 2020) local *π*_S_ = 0.011 (above) giving a scaled mutation rate of 1.53 × 10^−7^; estimated recombination rate of 1.655 × 10^−7^ and scaled recombination rate of 4.52 × 10^−6^. Three different migration rates were used, to span a range of degrees of population structure: *m *=* *0.00898 (i.e., *F*_ST_ = 0.05, similar to European *D. melanogaster*); *m *=* *0.00142 (*F*_ST_ = 0.26, equal to the overall mean seen in *D. magna*); and *m *=* *0.000158 (*F*_ST_ = 0.76, 3-fold greater than the mean). Three different rates of sexual reproduction were used, to bracket the range that is credible for *D. magna*: no clonality (all individuals result from sexual reproduction), daphniid-like clonality (7 of 8 generations 99% clonal individuals, 1 in 8 generations fully sexual), and extreme clonality (79 of 80 generations 99% clonal individuals, 1 in 80 generations fully sexual). We confirmed that observed local *π*_S_ and *F*_ST_ were close to the values predicted by theory for unconstrained sites. As our primary objective was to establish whether the estimate of $\omega_{a}$ and high estimate of the proportion of weakly deleterious nonsynonymous polymorphisms, obtained from *D. magna* were an artifact of our sampling strategy and population structure, we chose selection parameters based on those previously used to reflect *D. melanogaster* (Campos and Charlesworth 2019), which displays high rates of adaptive protein evolution, and fewer weakly deleterious amino-acid variants. Deleterious mutations were drawn from a gamma distribution, with a mean 2*N*_e_*s* of −2,000, and a shape parameter of 0.3 (scaled mean *s* of −0.056), and beneficial mutations all had 2*N*_e_*s* of 250 (scaled *s *=* *0.007). Both beneficial and deleterious nonsynonymous mutations were additive in their effects at a locus (no dominance), and 0.022% of nonsynonymous mutations were beneficial. For each of the nine sex and migration parameter combinations we ran 20 independent simulations for one million generations, and we only analyzed fixations and polymorphisms that arose after genetic diversity had equilibrated (supplementary figs. 1–3, Supplementary Material online). For each parameter combination, all mutations were combined across replicates to provide the estimates presented. We analyzed these simulations with three different sampling strategies, 1) one diploid per deme, 2) an equal-effort approach of 36 diploids from a single deme, and 3) 360 diploids sampled evenly across demes. The SLiM script and R code necessary to parse the output is provided as supporting material, Supplementary Material online.

## Supplementary Material

Supplementary data are available at *Molecular Biology and Evolution* online.

## Supplementary Material

msac048_Supplementary_Data
