# Supplementary material for: Population-Genomic Analysis Identifies a Low Rate of Global Adaptive Fixation in the Proteins of the Cyclical Parthenogen Daphnia magna
Source: Mol Biol Evol. 2022 Mar 4;39(3):msac048. doi: 10.1093/molbev/msac048 (PMC8963301; doi:10.1093/molbev/msac048)
Supplement: msac048_Supplementary_Data [file msac048_supplementary_data.zip › Fields_etal_genomewideSelection_Revision_22112021_Suppl.docx]

Supplemental Material

**Supplementary Table 1.** Alternative MK models fitted using the ML approach of Welch (2006)

We fitted a large number of alternative MK models using the approach of Welch (2006), in which genes can vary in the level of constraint (called *f*) and the population mutation rate (θ), as well as varying in α. We examined all combinations of models in which α was fixed at zero, had a single estimated value across genes, or had a different value in each functional class of genes, and models in which θ and *f* each took a single value, varied by locus, or varied by gene class. The two best-fitting models (according to the Akaike information criterion) are marked in bold. For models in which there were five discrete gene classes, the order is given at the bottom of the table (Control/Other, Immune, RNAi, Male, Female). For models in which θ and *f* varied by locus, the five reported values are 95% range, the interquartile range, and the median across loci (italic).

**Supplementary Table 2.** Results from MultiDFE estimate of $\alpha$ and $\omega_{a}$. Inputs for MultiDFE are the same as those used for DFE-alpha, including a folded SFS and divergence estimates of *D. magna* compared to *D. similis*. The best-fitting model (according to the Akaike information criterion) is marked in bold.

| Model | $\log L$ | K | $\Delta\mathrm{AIC}$ | Akaike Weight | $N_{E}s$ | | | | | $\bar{N_{E}s}$ | $\bar{u}$ | $\alpha$ | $\omega_{a}$ |
| --- | --- | --- | --- | --- | --- | --- | --- | --- | --- | --- | --- | --- | --- |
|  |  |  |  |  | [0-0.1) | [0.1-1) | [1-10) | [10-100) | $\geq$100 |  |  |  |  |
| lognormal | -3720402 | 2 | 130 | 0 | 0.21 | 0.00 | 0.00 | 0.00 | 0.79 | 0.000 | 0.208 | -0.044 | -0.009 |
| gamma | -3720469 | 2 | 264 | 0 | 0.19 | 0.01 | 0.01 | 0.01 | 0.78 | -1.679 | 0.200 | -0.004 | -0.001 |
| beta | -3724429 | 2 | 8184 | 0 | 0.20 | 0.00 | 0.00 | 0.00 | 0.80 | -0.803 | 0.197 | 0.014 | 0.003 |
| six-fixed-spikes | -3724429 | 5 | 8178 | 0 | 0.20 | 0.00 | 0.00 | 0.00 | 0.80 | -0.803 | 0.197 | 0.014 | 0.003 |
| one-spike | -3841575 | 1 | 242478 | 0 | 0.00 | 0.00 | 1.00 | 0.00 | 0.00 | -0.019 | 0.018 | 0.911 | 0.182 |
| two-spike | -3720371 | 3 | 66 | 0 | 0.00 | 0.21 | 0.00 | 0.00 | 0.79 | -0.001 | 0.187 | 0.065 | 0.013 |
| three-spike | -3720371 | 5 | 62 | 0 | 0.00 | 0.21 | 0.00 | 0.00 | 0.79 | -78.785 | 0.187 | 0.065 | 0.013 |
| four-spike | -3720371 | 7 | 58 | 0 | 0.00 | 0.21 | 0.00 | 0.00 | 0.79 | -0.001 | 0.187 | 0.065 | 0.013 |
| five-spike | -3720371 | 9 | 54 | 0 | 0.00 | 0.21 | 0.00 | 0.00 | 0.79 | -0.001 | 0.187 | 0.065 | 0.013 |
| one-step | -3788033 | 1 | 135394 | 0 | 0.00 | 0.03 | 0.29 | 0.68 | 0.00 | -0.100 | 0.027 | 0.866 | 0.173 |
| two-step | -3720340 | 3 | 4 | 0.12 | 0.08 | 0.13 | 0.00 | 0.00 | 0.79 | -0.001 | 0.186 | 0.065 | 0.013 |
| **three-step** | **-3720340** | **5** | **0** | **0.85** | **0.00** | **0.21** | **0.00** | **0.00** | **0.79** | **-0.001** | **0.186** | **0.068** | **0.014** |
| four-step | -3720346 | 7 | 8 | 0.02 | 0.08 | 0.13 | 0.00 | 0.00 | 0.79 | -5.273 | 0.186 | 0.065 | 0.013 |
| five-step | -3720348 | 9 | 8 | 0.02 | 0.11 | 0.10 | 0.00 | 0.00 | 0.79 | -4.254 | 0.193 | 0.034 | 0.007 |

**Supplementary Figure 1**. The life cycle of *D. magna*.

**Supplementary Figure 2.** AsymptoticMK estimate of genome-wide α from *D. magna.* The two vertical blue lines show the limits of the frequency cutoff interval used for fitting. Points indicate binned values of α(x), estimated according to Equation 2 in (Haller and Messer 2017a). The solid red curve shows the fitted αfit(x). The dashed red line shows the estimate of α_asymptotic,_ obtained from the fitted function according to Equation 3 in (Haller and Messer 2017a). The gray band indicates the 95 % CI around this α_asymptotic_ estimate. The dotted gray line shows the estimate of α_original_, obtained from the original (non-asymptotic) McDonald–Kreitman (MK) test, for comparison.

**Supplementary Figure 3: Convergence of** $\boldsymbol{\pi}$ **during the simulation**. Plots show mean within-deme $\pi_{A}$ (red) and $\pi_{S}$ (blue) across the 20 simulation replicates (pale lines) and their mean (dark line) over the course of 1million generations. The three rows show different levels of population structure (labelled by expected $F_{ST}$ ), and the three columns show different rates of asexual reproduction. We used pairwise differences within a single sampled individual to estimate π, and estimated effective population size (found by dividing local $\pi_{S}$ by 4$\mu$) is given in the corner of each plot. Note that diversity (and thus $N_{e}$) reaches its equilibrium value after ca. 350 million generations, but can be substantially reduced below its expected value by selection across the linked loci and by inbreeding through intra-clone mating. The impact of asexuality on the efficacy of selection can be seen in the change in the relative value of $\pi_{A}$ compared to $\pi_{S}$.

**Supplementary Figure 4: Convergence of** $F_{IT}$ **during the simulation**. Plots show $F_{IT}$, the total inbreeding due to combined population structure and local inbreeding, across the 20 simulation replicates (pale lines) and their mean (dark line) over the course of 1 million generations. The three rows show different levels of population structure (labelled by expected $F_{ST}$), and the three columns show different rates of asexual reproduction. $F_{IT}$ was estimated using the diversity of unconstrained sites as ${(\pi}_{Total}-\pi_{Local}$)/$\pi_{Total}$ total, where $\pi_{Local}$ was the mean across populations of the pairwise diversity within a single diploid individual. As expected, under random mating within demes (left column) $F_{IT}$ is identical to expected $F_{ST}$, because $F_{IS}$ is zero. $F_{IT}$ is slightly elevated relative to $F_{ST}$ as the rate of asexuality increases, because intra-clone mating can occur (i.e. genetic selfing).

**Supplementary Figure 5: Stabilisation of the fixation rate during the simulation**. Plots show the accumulated number of fixations over the course of 1 million generations for unconstrained (blue), deleterious (red) and beneficial (green) mutations across the 20 simulation replicates (pale lines) and their mean (dark line). The three rows show different levels of population structure (labelled by expected $F_{ST}$), and the three columns show different rates of asexual reproduction. The fixed deleterious alleles are those that have very small selection coefficients, i.e are behaving effectively neutrally, and the impact of asexuality on the efficacy of selection and $N_{e}$ can be seen in the change in the relative rate of fixation of weakly deleterious alleles relative to unconstrained alleles. Note that the rate of fixation of all mutation types is constant after ca. 350 million generations.

**Supplementary Figure 6: Equilibrium site frequency spectrum in an island model with selection and asexuality.** The folded site frequency spectrum is shown for unconstrained (synonymous; blue) and potentially selected (non-synonymous; red) sites, based on a simulation of 36 demes, and sampling a single diploid individual from each deme. Columns (left to right) increasing rates of asexuality, and rows (top to bottom) show increasing rates of migration. The greater skew at selected sites caused by selection against deleterious variants can be seen in all panels, and the ‘stepped’ distribution caused by sampling single diploid individuals from highly differentiated posts can be seen in the top row.

**Supplementary Figure 7: Inferred DFE and** $\boldsymbol{\alpha}$**when one individual is sampled from each population**. Each plot shows (left panel) the DFE of deleterious mutations and (right panel) α, the proportion of selectable fixations that were adaptive, when sampling one diploid individual from each of 36 demes. The DFE is shown as the proportion of mutations falling in classes of 2$N_{e}s$ in the ranges [0,-1), [-1,-10), [-10,-100) and $\underline{<}$-100. The bars show the true (simulated) values, and the estimates obtained by DFE-alpha, multi-DFE, and asymptotic-MK. For Multi-DFE, the model preferred by AIC is given where ‘st’ indicates a step distribution and ‘sp’ indicates a spike distribution, followed by the number of steps or spikes. Note that, for low levels of structure and asexuality all methods perform reasonably well when sampling one diploid individual per deme, although Multi-DFE fails to recover the true (gamma) distribution of the DFE.

**Supplementary Figure 8: Inferred DFE and alpha when all individuals are sampled from a single population**. Plots are as described for supplementary figure 7. Note that, for low levels of structure and asexuality, all methods perform similarly to the sampling strategy in supplementary figure 7. However, performance is slightly poorer than seen for a single individual per deme, and estimates of $\alpha$ are more strongly downwardly biased, especially when there are high levels of asexuality. Again, Multi-DFE fails to recover the true (gamma) distribution of the DFE.

**Supplementary Figure 9: Inferred DFE and** $\boldsymbol{\alpha}$ **when many individuals are sampled from each of many populations**. Plots are as described for supplementary figure 7. Note that, for low levels of structure and asexuality, all methods perform similarly to the sampling strategy in supplementary figure 7. However, performance is slightly poorer than seen for a single individual per deme, and estimates of $\alpha$ are more strongly downwardly biased, especially when there are high levels of asexuality. Again, Multi-DFE fails to recover the true (gamma) distribution of the DFE.

**Supplementary Figure 10.** **Distribution of** $\boldsymbol{\omega}_{\boldsymbol{a}}$**resulting from random samples of control gene set.** A) Random sample of 50 genes, B) random sample of 100 genes, and C) random sample of 1000 genes. Blue vertical line indicates $\omega_{a}$ estimate from full control gene dataset.
